# Nanoparticle-Free 3D-Printed Hydrophobic Surfaces for Ice Mitigation Applications

**DOI:** 10.3390/molecules30153185

**Published:** 2025-07-30

**Authors:** Ranim Zgaren, Maryam Hosseini, Reza Jafari, Gelareh Momen

**Affiliations:** 1 Department of Applied Sciences, University of Québec in Chicoutimi (UQAC), 555, Boul. de l’Université, Chicoutimi, QC G7H 2B1, Canada; ranim.zgaren@gmail.com (R.Z.); hmmaryamsa@etu.uqac.ca (M.H.); gelareh_momen@uqac.ca (G.M.); 2 Department of Mechanical Engineering, National Engineering School, Monastir 5035, Tunisia; 3 Department of Aerospace Engineering, École de Technologie Supérieure (ETS), Montréal, QC H3C 1K3, Canada

**Keywords:** anti-icing surfaces, microstructure, nanoparticle free, hydrophobicity, LCD 3D printing

## Abstract

Ice accumulation on exposed surfaces presents substantial economic and safety challenges across various industries. To overcome limitations associated with traditional anti-icing methods, such as the use of nanoparticles, this study introduces a novel and facile approach for fabricating superhydrophobic and anti-icing microstructures using cost-effective LCD 3D printing technology. The influence of diverse pillar geometries, including square, cylindrical, hexagonal, and truncated conical forms, was analyzed to assess their effects on the hydrophobic and anti-icing/icephobic performance in terms of wettability, ice adhesion strength, and icing delay time. The role of microstructure topography was further investigated through cylindrical patterns with varying geometric parameters to identify optimal designs for enhancing hydrophobic and icephobic characteristics. Furthermore, the effectiveness of surface functionalization using a low surface energy material was evaluated. Our findings demonstrate that the synergistic combination of tailored microscale geometries and surface functionalization significantly enhances anti-icing performance with reliable repeatability, achieving ice adhesion of 13.9 and 17.9 kPa for square and cylindrical pillars, respectively. Critically, this nanoparticle-free 3D printing and low surface energy treatment method offers a scalable and efficient route for producing high-performance hydrophobic/icephobic surfaces, opening promising avenues for applications in sectors where robust anti-icing capabilities are crucial, such as renewable energy and transportation.

## 1. Introduction

In a world where the challenges of frost and water accumulation affect critical sectors such as aerospace, energy, and transportation, designing surfaces capable of resisting and limiting frost and moisture formation is an increasingly important area of scientific investigation [1,2]. Inspired by the remarkable water-repellent and self-cleaning properties of natural surfaces such as the lotus leaf and Salvinia [3], this surface mimics their inherent resilience. These natural examples have inspired extensive research efforts aimed at replicating their properties, leading to significant progress in the development of artificially engineered super-repellent surfaces. Consequently, natural systems such as lotus leaves, focusing on superhydrophobic surfaces, have been investigated in recent years. These leaves illustrate the “lotus effect” [3], where water droplets take a spherical shape and roll easily, carrying impurities. This ability results from low-surface-energy micro/nanostructures that create an air cushion and minimize contact with the liquid. Artificial superhydrophobic surfaces are inspired by this principle by integrating hierarchical roughness and hydrophobic materials [4,5]. To replicate these natural microstructures, different approaches such as lithography [6], anodization [7], laser or chemical etching [8], as well as plasma or templating methods, have been employed to enhance air entrapment at the microscopic scale. Although these methods allow good control over the generation of microstructures, they often require multiple steps to achieve the final patterning. However, it is not always possible to clearly distinguish between these approaches because they are sometimes used in combination. Liu et al. fabricated the superhydrophobic surfaces by designing and fabricating SiO_2_ mushroom-like doubly re-entrant structures capable of repelling even very low surface energy liquids, without the use of a hydrophobic coating [9].

The use of 3D printing represents a major technological development in making custom and complicated structures, opening exciting perspectives for the design of innovative superrepellent surfaces, especially inspired by nature [10,11,12]. In recent years, a variety of 3D printing techniques, such as stereolithography (SLA) [13], digital light processing (DLP) [14], fused deposition modeling (FDM) [15], and two-photon polymerization [16], have been employed to successfully fabricate superhydrophobic materials. However, several geometric complexities exist in printing processes depending on the type of technology used. For example, FDM is affordable and accessible, but the objects obtained have visible layer lines [17]. Stereolithography (SLA) offers high accuracy and can generate complex structures, but resin processing can be difficult [18]. Other techniques, such as selective laser sintering (SLS) and digital light deposition (DLP), allow for high resolutions but may be too expensive or have hardware compatibility limitations [19,20,21,22]. The study of bio-inspired microstructure surfaces has highlighted the pillar-like structure in which the geometric parameters of pillars are related to their wettability properties. It was shown that tuning these parameters, such as pillar height, diameter, and spacing, is essential to maximize superhydrophobicity [23]. Using 3D printing techniques allows for precise control of these geometric parameters. Joyee and Pan fabricated surfaces with tunable wettability by adjusting the re-entrant angle and spacing parameters to achieve contact angles greater than 150°, demonstrating the potential of these techniques for functional surfaces [24]. In research using 3D printing techniques, such as DLP, the height of the pillars increased, which increased the apparent contact angle to further enhance the water repellency. Hu et al. fabricated microstructures inspired by springtail cuticles, demonstrating that with heights higher than 150 µm, superhydrophobicity is enhanced. However, if the pillars become too high, it can reduce the mechanical stability of the structure, especially for 3D printing based on photopolymer resins [25]. Deng Xu et al. highlighted the critical balance between hydrophobicity and structural stability by designing dual-scale structures with micro-level shielded frames housing nanostructures. Increasing the pillar diameters to an optimal size improves robustness without significantly compromising hydrophobicity, especially when combined with high aspect ratios [26]. Zhang et al. created samples with microscale square pillars by photolithography and dry etching and demonstrated that pillar spacing has a significant impact on the air-trapping mechanism. They found that spacings between 100 and 200 µm maximize water repellency while preserving the structural integrity of the microstructure. Too close a spacing may cause a transition to the Wenzel state due to insufficient air gaps [27]. All the mentioned studies have mainly focused on superhydrophobic surfaces, examining how surface topography and variations in geometric parameters affect wettability. Despite these advancements in creating highly repellent surfaces, the design principles for optimal superhydrophobicity do not always align with those required for effective anti-icing [28]. While micro- and nano-scale textures enhance water repellency by promoting droplet roll-off, these same features can inadvertently serve as nucleation sites for ice formation or increase mechanical interlocking, thereby elevating ice adhesion. Thus, achieving a surface with both robust superhydrophobic and anti-icing properties continues to be a major challenge in research and development [29]. It should be noted that while 3D printing provides notable advantages for the rapid prototyping of microstructured surfaces used in anti-icing applications—particularly for replicating complex, nature-inspired geometries—it is important to recognize that, like many early-stage technologies, it also comes with certain limitations. One major constraint is the resolution limit, which can restrict the minimum achievable feature size, such as micropillar diameter and spacing [30]. Additionally, the scalability of high-resolution 3D printing to large-area surfaces remains challenging due to the time-consuming nature and limited build volume of most systems [31]. Therefore, the need for post-processing steps (e.g., surface coating or functionalization) should still be considered when applying these techniques to practical anti-icing surfaces. While a few studies have investigated the anti-icing properties of pillar-shaped structures, 3D printing methods have not been used to fabricate them. Hu et al. fabricated micro-cubic pillar structures using plasma etching technology and investigated their anti-icing performance [32]. There is only one study that has examined 3D-printed structures for their anti-icing properties, focusing mainly on maintaining mechanical properties by the addition of siloxane components [33]. However, most investigations have focused on microstructures developed through lithography or other complex methods, with limited direct studies on how geometric parameters affect the ice-repellent properties of 3D printed structures. Given its simplicity and versatility, 3D printing presents a promising alternative for fabricating such surfaces, especially when combined with hydrophobic resins, which could further enable the development of superhydrophobic and icephobic functionalities.

In the present study, LCD 3D printing (liquid crystal display), as a relatively new method, was utilized as a facile and cost-effective approach to design and fabricate superhydrophobic surfaces with integrated anti-icing properties, achieved without the use of nanoparticles. Compared to other 3D printing techniques, the LCD method offers key advantages for microstructured surface fabrication, including high resolution, lower cost, faster layer exposure, and uniform light distribution, enabling efficient production of regular features like micropillars for surface engineering applications. The primary objective of this work is to examine how the microstructural design of 3D-printed surfaces influences their anti-icing performance. Various pillar geometries—including square, cylindrical, hexagonal, and truncated conical—were studied for their influence on surface wettability. Subsequently, the effects of geometric parameters such as diameter, height, and spacing between pillars were systematically evaluated. Finally, the printed surfaces were chemically modified with fluorosilane to further enhance water repellency and reduce frost accumulation, which was primarily induced by the roughness created through 3D printing. This dual approach, combining roughness induced by microstructural design with surface chemistry, offers a promising pathway for creating functional materials suited to harsh, icy environments.

## 2. Results and Discussion

### 2.1. Morphological Evaluation of Microstructures

In this study, for printing microstructures, a commercial acrylate-based resin was employed. Different geometrical forms of the pillars, cylindrical, square, hexagonal, and truncated conical, were evaluated. These shapes are inspired by natural structures that are well known for their ability to repel water. Cylindrical pillars, for instance, are commonly found in literature due to their effectiveness in trapping air and reducing ice adhesion [26]. They strike a practical balance between hydrophobic performance and ease of fabrication using 3D printing. Truncated conical pillars mimic morphologies observed on insect wings and enhance the lotus effect by increasing air-trapping regions [34]. Square and hexagonal pillars were selected for their geometric regularity and spatial efficiency, which promote effective droplet drainage and reduce mechanical anchoring of ice [35]. This diversity in geometry allows for a comparative analysis of their influence on the superhydrophobic and anti-icing performance of the printed surfaces. The pillar dimensions were selected based on previous studies highlighting their effectiveness in modulating surface wettability and anti-icing behavior. As reported by Zhang et al. [27], air entrapment is optimized within a spacing range of 100–200 µm, while avoiding transition to the Wenzel regime. Deng Xu et al. and Wang et al. further emphasized that aspect ratios between 1:1 and 1:4 enhance hydrophobicity while preserving mechanical stability [26,35]. Our designs reflect this range, with pillars spanning from 1:1 (120 µm height and diameter) to 4:1 (480 µm height, 120 µm diameter). To ensure uniform air-pocket formation and isolate the effect of size scaling, pillar diameter and spacing were kept equal within each sample (M80, M100, and M120). Taller structures (H360 and H480) were introduced to specifically assess the influence of vertical elevation on droplet mobility and ice adhesion.

First, a control sample without any surface structure was initially printed and compared to the pillar-shaped structures fabricated using a 3D printer, to evaluate their influence on wettability and anti-icing properties. The control sample was characterized by optical microscopy, profilometry, and contact angle determination (Figure S1). The profilometry analysis did not show any surface roughness on the control sample. As expected, due to the hydrophilic groups of acrylate-based resin on the surface and the lack of surface roughness, the contact angle was measured to be 45°.

**Figure 1:**
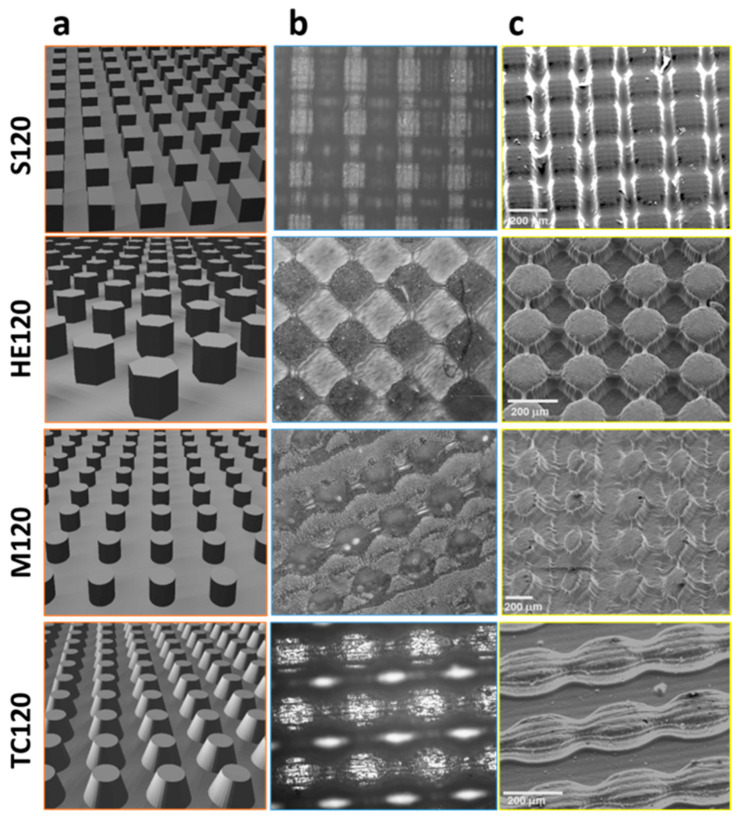
(**a**) Solid work design, (**b**) optical microscope (100×), and (**c**) SEM images of the S120, HE120, M120, and TC120 microstructures, taken at a tilting angle of 35°.

Initially, to examine the effect of pillar morphology, microstructures with different pillar shapes, square (S120), cylindrical (M120), hexagonal (HE120), and truncated conical (TC120), were printed and compared in terms of WCA and SA, all at a scale of 120 microns. Then, the effect of pillar size and height was investigated as Supplementary Data and incorporated into the analysis. Morphology of the printed microstructures was investigated through optical and electron microscopy. The images related to the solid work design, optical microscope, and SEM are shown in Figure 1. The results showed that the pillars were accurately printed and consistent with the corresponding geometry. The actual dimensions of the printed samples are shown in Table S1. Optical microscopy and SEM images effectively confirmed the morphology of the pillars. Moreover, a closer evaluation of the images revealed a variation range of about 10–20 μm in the selected diameters and spacings. The results showed some interconnections between the pillars within a scale of 20 to 40 microns. This probably can be related to the fact that most of these pillars were aligned linearly, thus allowing more resin diffusion between them during the curing process. Furthermore, the viscosity of the resin is another factor to consider; this may hinder proper flow in narrow spaces between pillars, causing bonds between them in the curing process. The printed M120 and HE120 showed geometric compliance, with enough printing accuracy, and with small connections.

### 2.2. Topographical Study of Microstructures

**Figure 2:**
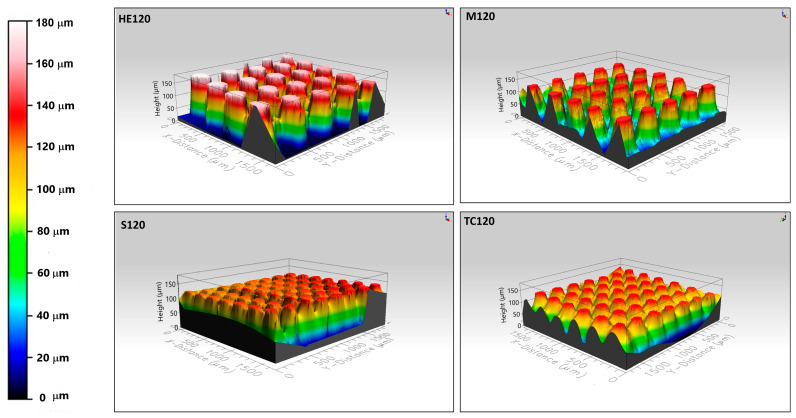
The 3D profile images of S120, HE120, M120, and TC120 microstructures.

A three-dimensional (3D) profilometer was applied to provide precise information on the geometry, surface roughness, and height consistency of the pillars. Figure 2 shows the 3D profile images of the microstructures with different pillar shapes, indicating the corresponding surface structure. Moreover, the surface height maps evidenced that the heights were measured with a small error, with proper consistency compared to expected designs.

### 2.3. Influence of Geometric Parameters and Surface Functionalization on Wettability

**Figure 3:**
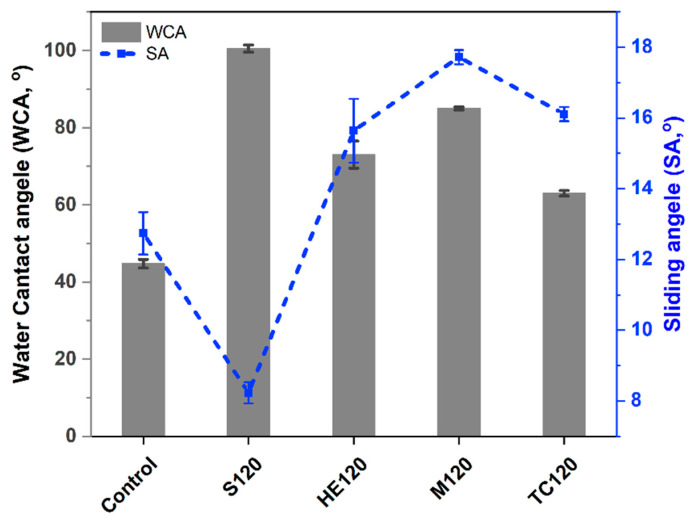
Wetting properties of S120, HE120, M120, and TC120 microstructures, WCA and SA, before FAS modification.

The wettability of the printed microstructures was evaluated through measurements of WCA and SA as well. Initially, the influence of the pillar shapes was investigated, as shown in Figure 3. To ensure the accuracy of our results, each test was repeated for each microstructure. For the control microstructure, a contact angle of 45° was obtained. For S120 with a square pillar, a contact angle of 100.5° was generally associated with strong water repellency, suggesting a hydrophobic surface. The low sliding angle of 8.3° indicates minimal adhesion and high-water mobility, highlighting the surface’s strong water-repellent properties. In comparison, TC120, with conical pillars, presented a WCA of 62.5°, revealing less pronounced water repellency. Higher SA and lower WCA values of TC120 as compared to S120 could be related to more significant interactions between water and the surface, which might allow for partial wetting rather than stable droplet formation. Similarly, for the M120 microstructure, a contact angle of 85° suggests moderate water repellency. The SA of 17.7° indicates increased water adhesion, which may contribute to partial wettability and reduced droplet mobility. The results obtained for the hexagonal pillars indicate another wetting dynamic. When the water drops contact the surface, first a WCA of 73° was observed, but it spread after about 10 s, indicating that the overall surface properties were not hydrophobic. Consequently, hexagonal pillars may fail to exhibit superhydrophobic behavior. Significant differences in the wettability characteristics were shown by analyzing the results, highlighting the importance of pillar geometry in the creation of hydrophobic surfaces.

**Figure 4:**
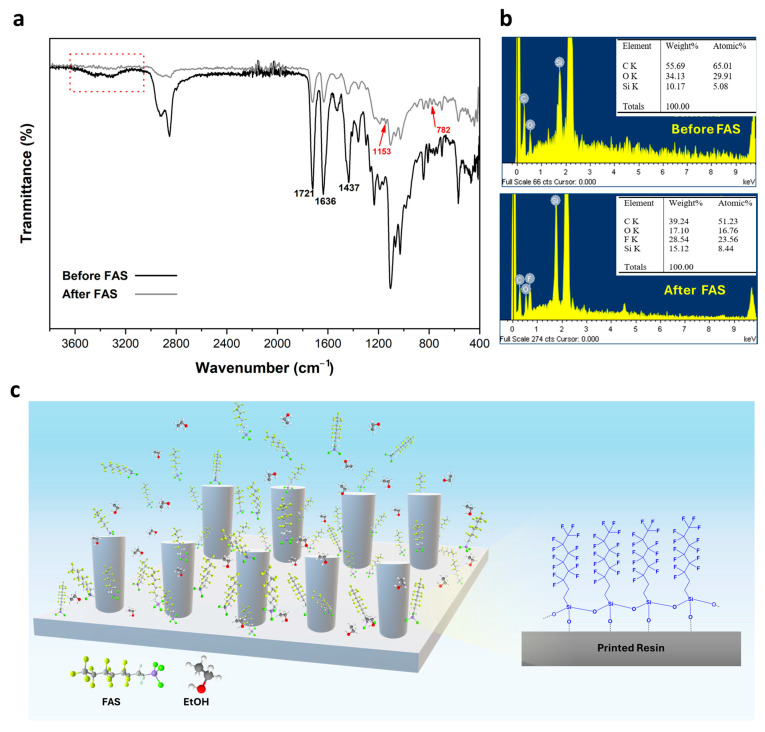
(**a**) FTIR spectra and (**b**) EDS analysis of M120 before and after surface functionalization with FAS; (**c**) schematic illustration of FAS modification of microstructures.

Fabricated microstructures with different pillar shapes were evaluated in terms of wettability by measuring the contact angle and sliding angles. Moreover, to improve the performance of the printed samples, all of them were functionalized with FAS, and all the evaluations were performed before and after surface modification. To this end, first, the printed microstructures were subjected to interaction with FAS and characterized. The prepared samples were characterized via FTIR and EDS analyses. Figure 4a shows the infrared spectra of M120 before and after functionalization with FAS. As previously mentioned, commercial acrylate-based resins were used to print the microstructures, and the characteristic bands of acrylate are well-represented in the corresponding spectrum. The absorption bands that appeared between 3200–3600 cm^−1^ are related to the stretching vibrations O-H bonds. Moreover, the stretching vibrations of aliphatic C-H were located at 2926 and 2851 cm^−1^. The stretching vibrations of the ester C=O bond and C=C bond of the acrylate appeared in 1721 and 1636 cm^−1^, respectively [36,37]. As shown, after the surface functionalization of the printed microstructure with FAS, the vibration pattern of the resin remained. In addition, two new peaks appeared at 1153 and 782 cm^−1^, which could be assigned to stretching vibrations of C-F (perfluorinated group -CF_3_, -CF_2_-) and Si-Cl (trichlorosilane) bonds in FAS, respectively [38]. Also, the decrease in the intensity of the resin absorption bands and their broadening, especially in the region of 3000 to 3500 cm^−1^ related to the hydroxyl groups, could be attributed to the covalent interaction of the resin surface with FAS. Moreover, as reported by Catterton et al. [39], a notable decrease in the carbonyl group at 1721 cm^−1^ could be another sign of possible covalent interaction between oxygen-containing groups on the printed structures and the silane groups of FAS (Figure 4c), which is consistent with the wettability results, discussed in future parts.

Further investigation was carried out using surface elemental analysis (Figure 4b). According to the results of EDS analysis after the surface functionalization with FAS, the atomic percentage of fluorine element on the surface could be obtained at about 28%, demonstrating the successful surface functionalization of the printed microstructures with FAS. Figure 4c shows the schematic illustration of the surface modification of pillar-shaped microstructures.

**Figure 5:**
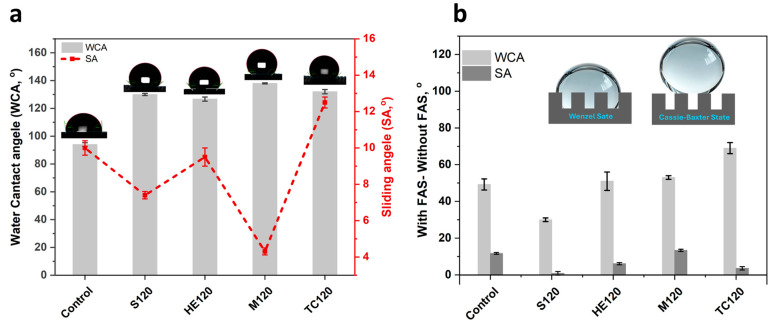
(**a**) Wetting properties of S120, HE120, M120, and TC120 microstructures, WCA and SA, after FAS modification; (**b**) differences in WCA and SA values before and after FAS modification.

The WCA and SA values of the printed microstructures after surface functionalization with FAS are shown in Figure 5a. The results showed that the functionalization process led to a significant increase in the contact angle of the microstructures. In all samples, an increase in WCA and a decrease in SA could be observed. Except for the probable covalent bond between FAS and microstructure surface, which has already been explained, fluoroalkyl chains are inherently hydrophobic, meaning they repel water and prefer to associate with other hydrophobic substances. The hydrophobic portions of the acrylate resin (e.g., alkyl groups from monomers like butyl acrylate) can interact favorably with the fluoroalkyl chains through van der Waals forces and hydrophobic interactions, further contributing to their compatibility. These fluoroalkyl groups tend to orient themselves outward, forming a self-assembled monolayer on the surface [40]. This orientation results in a highly ordered structure that minimizes surface energy and significantly increases contact angle. The result is a fluorinated surface layer that exhibits excellent hydrophobicity, which effectively lowers the surface energy and improves the wettability. Figure 5b represents the differences obtained by subtracting the WCA and SA values of pre-modification from the post-modification samples with FAS. A significant improvement of approximately 50° could be observed for the WCA for both cylindrical and hexagonal pillars (M120 and HE120), indicating a partial transition from the Wenzel state to the Cassie–Baxter state, which decreases the contact of the surface with water [41]. For the truncated conical microstructure, an increase of more than 60° for the contact angle could be observed. This could be attributed to the uniform distribution of the FAS functionalization, which creates a chemical barrier that could trap air pockets, reinforcing a stable Cassie–Baxter regime and limiting ice anchoring points [34]. Profilometry results indicated that the highest height of the interconnections was related to this sample, which may facilitate a more uniform distribution of the FAS coating and consequently lead to a greater increase in WCA value. This highest interconnection induced mild anisotropic behavior in this sample. The sliding angle (SA) of TC120 was measured in both directions, and a difference of approximately 3° was observed between the two directions. As expected, in the direction perpendicular to the aligned structures, the water droplet encountered greater resistance, compared to the direction parallel to the structures [42]. It should be noted that following FAS functionalization, the contact angle for all samples became relatively similar. Since the square pillars have undergone fewer changes, it could be concluded that a substantial portion of the observed hydrophobicity originated from the inherent surface structure. A similar trend was observed for the sliding angle as well. The smallest change was related to S120, and the greatest one was related to M120.

**Figure 6:**
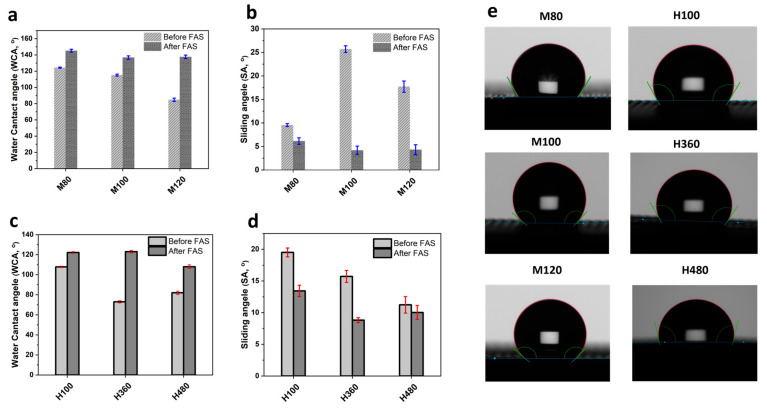
The influence of height (**a**,**b**) and size (**c**,**d**) of pillars on WCA and SA before and after FAS modification; (**e**) WCA images of M80, M100, M120, H100, H360, and H480 after FAS modification.

The changes in the geometric parameters for each selected geometry of the pillars influence the interactions between the surface and the water molecules. Therefore, the effect of two parameters, pillar size and pillar height, in cylindrical microstructures on WCA and SA was studied (Figure 6a–c). Figure 6a,b investigates how variations in pillar size impact the wettability characteristics of the surface before and after modification with FAS. The optical microscope and SEM images of M80 and M100 are also shown in Figure S2. Before FAS modification, the M80 microstructure showed the highest contact angle and lowest sliding angle, which comes from its inherent characteristics. After FAS functionalization, it could be seen that the M80 had reached the superhydrophobicity threshold. As shown in Figure 6a, by increasing the size of pillars, accompanied by an increase in both the spacing and height of the structures, a noticeable decrease in the contact angle could be observed. This behavior could be attributed to the deeper penetration of the droplet meniscus into the gaps between the pillars without touching the surface. Under these conditions, the wetting regime tends to shift from the Cassie–Baxter state to the Wenzel state, where more of the droplet surface meets the solid substrate. Consequently, the contact angle decreases. The increased spacing hinders effective air entrapment, thereby weakening the Cassie–Baxter state regime that is essential for achieving superhydrophobicity [43].

The influence of pillar height on wettability was also evaluated under constant pillar spacing and diameter (Figure 6c,d). The corresponding optical and SEM images are shown in Figure S3. Although increasing pillar height is generally expected to enhance surface roughness and promote higher contact angles [44,45], in the present study, a slight reduction in contact angle could be observed. This trend might be explained by the deeper penetration of the droplet meniscus into the taller interspaces, which facilitates increased solid–liquid contact and promotes a partial transition toward the Wenzel regime. In taller structures, particularly beyond 300 µm, the effectiveness of air entrapment between the pillars could be reduced due to the limited ability of the meniscus to remain suspended near the pillar tops [33]. Additionally, capillary forces along the vertical sidewalls become more dominant at these scales, further encouraging wetting and decreasing the apparent contact angle. These combined effects suggest that merely increasing height, without adjusting other geometrical parameters, may not always enhance hydrophobicity and can even promote wetting under certain conditions [35].

### 2.4. Study of the Anti-Icing Properties

#### 2.4.1. Ice Nucleation Delay Time

**Figure 7:**
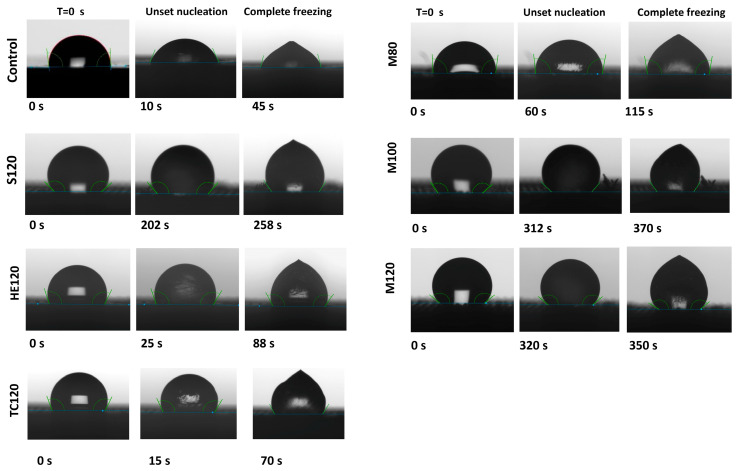
Freezing delay time for S120, HE120, TC120, M120, M100, and M80 microstructures.

The delay of the freezing process of water droplets on a surface constitutes the primary mechanism driving anti-icing effectiveness. In the freezing experiments, droplet solidification occurred in two stages: ice nucleation and complete freezing. Nucleation time, measured with a high-speed camera, was the interval from droplet deposition on a pre-cooled surface to the onset of rapid recalescence. After nucleation, complete freezing followed as ice grew throughout the droplet until it was fully solid, also tracked using high-speed imaging. The present research studied the impact of pillar morphology, particularly shape, diameters, and height, on the inhibition of ice formation (Figure 7). The wetting state between the solid surface and the liquid is essential in determining icing delay time. Figure 7 demonstrates that the printed microstructures featuring cylindrical and square-shaped pillars displayed the greatest delay in freezing time, highlighting the significant impact of surface morphology on anti-icing efficacy. According to Figure 5a, these samples showed relatively high WCA and the lowest SA compared to the other samples. These structures could probably enhance droplet mobility and prevent penetration into the texture, thereby prolonging freezing time [32]. For the microstructures with cylindrical pillars (M80, M100, and M120), the results revealed that the geometric dimensions of the pillars influence the anti-icing properties as well. M120 and M100 indicated much higher nucleation delay times as compared to M80. Increasing the pillar heights reduces the likelihood of penetration of the droplet into the pillars and can better sustain a Cassie–Baxter state by minimizing solid–liquid contact [43]. This effect is also linked to the entrapment of more air pockets underneath the water droplets, which acts as a thermal insulator, slowing down heat transfer and thus prolonging the ice nucleation time [46,47]. For the microstructures with cylindrical pillars (M80, M100, and M120), the results revealed that the geometric dimensions of the pillars influence the anti-icing properties as well. M120 and M100 indicated much higher nucleation delay times as compared to M80. Increasing the pillar heights reduces the likelihood of penetration of the droplet into the pillars and can better sustain a Cassie–Baxter state by minimizing solid–liquid contact [35]. This effect is also linked to the entrapment of more air pockets underneath the water droplets, which act as a thermal insulator, slowing down heat transfer and thus prolonging the ice nucleation time [39,40]. Analogous insights were reported by Liu et al., who emphasized that bio-inspired microstructures enhance anti-icing performance by trapping air pockets and promoting Cassie–Baxter wetting states [48].

#### 2.4.2. Ice Adhesion

**Figure 8:**
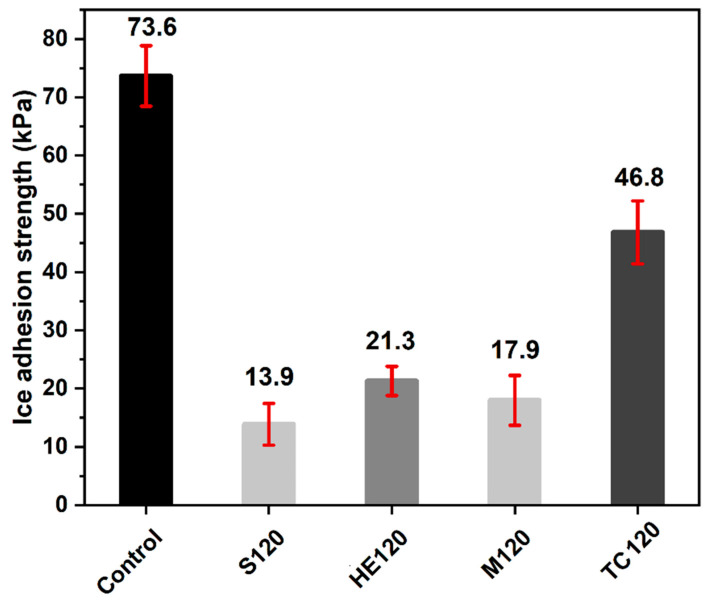
Ice adhesion strength of S120, HE120, M120, and TC120 microstructures.

The ice adhesion characteristics of all printed samples were examined to assess the anti-icing effectiveness associated with various pillar geometries (Figure 8). Notably, the square pillar (S120) and cylindrical pillar (M120) exhibited low ice adhesion strengths of 13.9 ± 3.4 kPa and 17.9 ± 4.2 kPa, respectively, indicating improved efficiency of the printed microstructures in preventing ice formation. In contrast, conical pillars demonstrated a significantly higher ice adhesion strength of 46.8 kPa. The push-off test analysis confirmed that the lowest ice adhesion forces are possessed by the surface with the lowest SA and highest WCA. This observation aligns with Zhang et al., who reported that micropillar arrays with optimized geometric parameters achieve ice adhesion as low as 10–20 kPa—attributable to minimized solid–ice contact area and enhanced Cassie–Baxter stability [49]. Specifically, when the SA exceeds 20°, the droplet anchoring before solidification increases, leading to greater detachment resistance. Interestingly, although certain structures, such as TC120, exhibited CAs greater than 120°, they still recorded high ice adhesion. This suggests that in some situations, the pillar shape will override the effect of the hydrophobicity and facilitate mechanical anchorage of the ice. The adhesion process is a complicated physical and chemical process with the interface interaction of Van Der Waals Forces, chemical bond cooperation, and microscopic mechanical connection. Hence, the optimal icephobic surface could be an optimum balance of low hysteresis, high superhydrophobicity, and the least mechanical contact with ice [50]. These findings align with previous studies indicating that while superhydrophobicity contributes to icephobicity, surface texture and structure play a more dominant role in determining ice adhesion strength [51,52]. Specifically, micro- and nano-structured surfaces that trap air pockets can reduce ice adhesion by minimizing the solid–ice contact area. However, if the surface structures allow for mechanical interlocking, ice adhesion can increase despite high water contact angles. Therefore, designing effective icephobic surfaces necessitates careful consideration of both surface chemistry and topography to achieve the desired anti-icing performance [32]. Additionally, the icing/de-icing cycling test in Figure S4 shows that the ice adhesion strength of the printed microstructures did not increase significantly after five cycles, indicating relative stability of FAS on the microstructure surfaces.

## 3. Experimental

### 3.1. Materials and Sample Preparations

Aqua Gray 4k, a commercial acrylate-based resin (Phrozen Tech Co., Ltd. Xiangshan District, Hsinchu City, Taiwan (R.O.C.), was employed for the 3D printing and has a density of 1.1 g/cm^3^, a viscosity of 150 cps, a tensile strength of 2 MPa, and an elongation of 7%. To get rid of any unsolidified resin residue and clean the surface, the samples are submerged in an isopropyl alcohol (Thermo Fisher Scientific Inc., Mississauga, Ontario, Canada) bath for two minutes after printing. They are then allowed to dry.

The printed microstructures are immersed in a solution of Trichloro(1H,1H,2H,2H-perfluorooctyl)silane, FAS (C_8_F_17_SiCl_3_: CF_3_(CF_2_)_5_CH_2_CH_2_SiCl_3_) (Thermo Fisher Scientific Inc., Mississauga, Ontario, Canada). This compound, from the family of perfluorinated silanes, makes the surfaces hydrophobic and anti-icing. The solution is prepared by mixing 0.5 mL of FAS in 10 mL of isopropyl alcohol to obtain a homogeneous solution. The samples are immersed for 10 min with sonication to ensure uniform distribution, then heated at 60 °C for 24 h for curing.

### 3.2. Fabrication of Microstructures

The printed microstructures were designed using SolidWorks 2018. We printed pillars of different geometric shapes on 15 × 15 mm^2^. The parameters include pillar diameter ($d_{pillar}(µm)$), a spacing between two pillars $\left(S_{pillar\_pillar}(µm\right)$), and a pillar height $\left(h_{pillar}\left(µm\right)\right)$. These microstructures were manufactured using a Phrozen Sonic Mini 4K 3D printer with LCD (liquid crystal display), (Phrozen Tech Co., Ltd. Xiangshan District, Hsinchu City, Taiwan (R.O.C.)) technology. This high-performance monochrome LCD printer offers a 4 K resolution of 3840 × 2160 pixels. It utilizes powerful UV LEDs beneath a 135 × 75 mm LCD screen to cure each resin layer with an accuracy of 35 microns on the X and Y axes. To optimize the 3D printing parameters, a set of experimental tests was conducted. For each test, only one parameter was adjusted while all the other parameters were kept constant, allowing for the identification of their influence on the print quality of the microstructures. This systematic approach led to the determination of an optimal configuration. The resolution is 3840 × 2160, and the lifting speed of the plate was optimized to 100 mm/min. The resolution of the modeling was also improved, with a layer height optimized to 0.03 mm, allowing a good compromise between precision and printing time (12 to 15 min). The exposure time of the bottom layer was set to 40 s to ensure solid adhesion without excess. These methodical adjustments significantly improved the quality of the flat surfaces and the accuracy of the printed microstructures.

The variety of the obtained textures arises from the choice of geometrical forms of the pillars: cylindrical, square, hexagonal, and truncated conical pillars. Sample names are assigned considering the morphology of the pillars. Square pillars are labeled by “S” and hexagonal ones by “HE”. For cylindrical pillars, the abbreviation “M” is used when the diameter, spacing, and height of the pillars are equal, and “H” is used when the diameter and spacing are equal but with different heights. Truncated cone-shaped structures are labeled as “TC”. Each abbreviation is followed by a number representing the geometric dimension’s measurement of the pillars, except for H, where the number represents only the height. Table 1 and Table 2 describe the geometric dimensions of the 3D-printed microstructures. 

### 3.3. Study of the Composition and Morphology of the Printed Microstructures

The surface of the printed microstructures was analyzed using scanning electron microscopy (SEM) (JSM-6480 LV SEM, JEOL, Tokyo, Japan) under a 20 kV accelerated voltage. The surface functional groups after FAS modification were identified using a Cary 630 FTIR spectrometer (Agilent, Santa Clara, CA, USA) in the attenuated total reflection mode and infrared range of 400–4000 cm^−1^.

### 3.4. Study the Topography of the Printed Microstructure

The dimensional characterization of the microstructures was performed using an optical profilometer (Profilm3D Filmetrics^®^, San Diego, CA, USA) to verify the topography of the printed surfaces, which integrated white light interferometry (WLI) to measure surface profiles at a precision of 0.05 μm. All three 3D images were obtained using the built-in software of the device (profilm), and no additional or external software was used for processing or visualization. The measurement included scanning the surfaces along a defined axis to measure the height differences of the structures. These measurements allow for the manufacturing method efficiency, surface roughness, and understanding of the morphology of the printed surfaces. These were then compared to wettability and ice adhesion performance to determine the impact of topographical characteristics on superhydrophobic and ice-phobic properties.

### 3.5. Study the Wettability of the Microstructures

To differentiate the resulting surfaces, the static contact angle (CA) and sliding angle (SA) of a demineralized water droplet are measured with a Kruss™ DSA100 goniometer (KRÜSS Scientific, Hamburg, Germany), maintaining a room temperature of 25 ± 0.5 °C. A water droplet (4 µL) is placed on the sample surface, and the average CA was calculated using the ADVANCE drop shape analysis software (version 1.16.0). To determine the sliding angle, a high-resolution camera is used to take images when the platform is tilted at an angle of up to 60°, and the sliding point is identified. The angle at this time is the sliding angle (SA), which is the critical point for the droplet to slide on the surface. The instrument is equipped with a cold chamber where the Peltier cooling controller allows it to reach –30 °C, with a control precision of 0.1 °C, to measure the contact angle at freezing temperatures and the freezing delay time.

### 3.6. Anti-Icing Properties

**Scheme 1:**
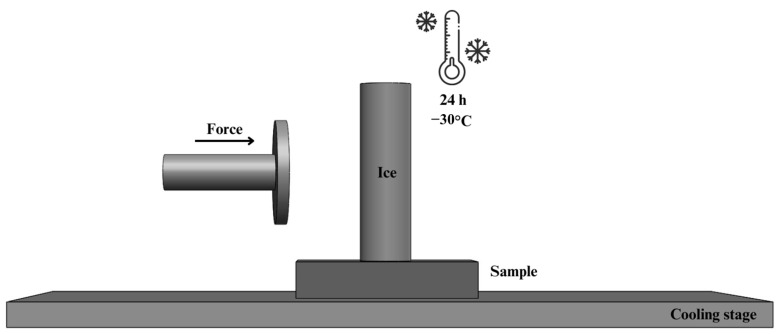
Schematic illustration of push-off test.

Anti-icing properties of the produced samples were determined using the push-off test. The test is a measurement of the shear stress at which the ice is peeled off from the surface. In this test, deionized water is sprayed in a cylindrical column of 8 mm diameter placed on the microstructure and then frozen at −30 ± 0.2 °C for 24 h before the testing. The microstructures were fixed on aluminum plates. With the initiation of a start with a computer-controlled remote interface, the screw is rotated at a constant velocity of 0.05 mm.s^−1^ such that the holder of the specimen is moved slowly towards the force gauge. The force gauge measures the shear force ten times per second until the detachment of the ice. Therefore, adhesion stress can then be measured considering the maximum force and the icing area. The lower the force required to detach the ice, the more resistant the surface is to ice. This test is repeated three times for each microstructure to ensure the reliability of the results (Scheme 1). Moreover, the samples underwent five icing/de-icing cycles to evaluate the stability of the FAS coating on the printed surfaces. Push-off tests were performed at the same surface location, all conducted at −10 °C. Additionally, the freezing delay time of various microstructures was examined at −20 °C and a relative humidity of 30 ± 5% using a Kruss™ DSA100 goniometer (KRÜSS Scientific, Hamburg, Germany), equipped with a Peltier plate inside a cold chamber. Anhydrous calcium sulfate desiccants were used within the chamber to minimize condensation and maintain stable humidity levels [53].

## 4. Conclusions

This study successfully demonstrated the feasibility of using a cost-effective LCD 3D printing technique for the fabrication of superhydrophobic microstructures with promising anti-icing properties. A range of pillar geometries—including cylindrical, conical, square, and hexagonal were successfully fabricated using LCD 3D printing and subsequently functionalized with FAS, resulting in improved water repellency and reduced ice accumulation. Among these microstructures, square pillars showed hydrophobicity with the highest WCA and the lowest SA before surface functionalization, which probably came from the inherent surface roughness. After FAS surface modification, in all samples, an increase in WCA and a decrease in SA were observed. The greatest difference in WCA was observed in the conical sample, whereas the square pillar sample exhibited the smallest change. A similar trend was observed for the sliding angle as well. The evaluation of cylindrical pillar structures showed that as the pillar size increases, the wetting mechanism transitions to the Wenzel state, leading to a reduction in the water contact angle (WCA) and an increase in the SA. Our results showed that the printed microstructures with cylindrical and square-shaped pillars exhibited the highest delay in freezing time. Moreover, it was confirmed that the ice adhesion can be reduced to only 13.9 and 17.9 kPa for square and cylindrical pillars, respectively, indicating more air pockets at the apparent solid-ice interface. In overall consideration of wettability, icing delay time, and ice adhesion, square and cylindrical pillars are proposed as promising microstructures for 3D printing in superhydrophobic and icephobic applications. These findings suggest that tailored microstructured surfaces fabricated via 3D printing offer a scalable and practical approach for developing advanced icephobic materials in industries where ice accumulation poses significant challenges. While this study provides valuable insights into the performance of different pillar shapes, future research could explore the long-term durability of these structures under harsh environmental conditions, investigate the influence of different functionalization materials, and optimize the printing parameters for large-scale production. Further investigation into the ice nucleation mechanisms on these surfaces could also provide a deeper understanding and lead to even more effective anti-icing design.

## Figures and Tables

**Table 1:** Nominal dimensions of microstructures with square, hexagonal, and truncated conical pillars.

Surface Morphology	Semple	$d_{pillar}(µm)$	$S_{pillar-pillar}(µm)$	$h_{pillar}(µm)$
Without pillars	Control	-	-	-
Square	S120	120	120	120
Hexagonal	HE120	120	120	120
Truncated cone	TC120	120	120	120

**Table 2:** Nominal dimension of cylindrical pillar microstructures.

Surface Morphology	Semple	$d_{pillar}(µm)$	$S_{pillar-pillar}(µm)$	$h_{pillar}(µm)$
Cylindrical	M80	80	80	80
	M100	100	100	100
	M120	120	120	120
	H100	120	120	100
	H360	120	120	360
	H480	120	120	480

## Data Availability

The data presented in this study are available on request from the corresponding author.

## Supplementary Materials

The following supporting information can be downloaded at: https://www.mdpi.com/article/10.3390/molecules30153185/s1, Figure S1: Microstructure control results (a) image of optical microscope (10X magnification, top view), (b) image of profilometer, (c) image of WCA measurement.; Figure S2. (a) SEM images M80 and M100 taken at a tilting angle of 35° (b) optical microscope images of M80 and M100 (100x), Figure S3. (a) SEM images H100, H360 and H480 taken at a tilting angle of 35° (b) optical microscope images of H100, H360 and H480 (100x), Figure S4. The ice adhesion strength of printed microstructures exposed to 5 icing/de-icing cycles obtained by push-off test, Table S1:Actual dimensions of printed microstructures.
