# Supplementary material for: Nanoparticle-Free 3D-Printed Hydrophobic Surfaces for Ice Mitigation Applications
Source: Molecules. 2025 Jul 30;30(15):3185. doi: 10.3390/molecules30153185 (PMC12348154; doi:10.3390/molecules30153185)
Supplement: Supplementary file 1 [file molecules-30-03185-s001.zip › molecules-3755078-supplementary.pdf]

Nanoparticle-Free 3D-printed Hydrophobic Surfaces for Ice Mitigation Applications

Ranim Zgaren[1], Maryam Hosseini [1], Reza Jafari [1]*, G. Momen [1, 2]

[1] *Department of Applied Sciences, University of Québec in Chicoutimi (UQAC), Quebec, Canada.*

[2] *Department of Aerospace Engineering, École de technologie supérieure (ETS), Quebec, Canada.*

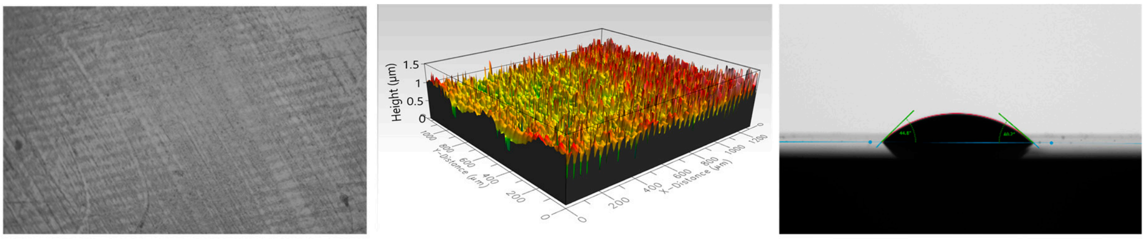

Figure S1: Microstructure control results (a) image of optical microscope (10X magnification, top view), (b) image of profilometer, (c) image of WCA measurement.

Table S1. Actual dimensions of printed microstructures.

**Surface morphology**	**Semple**	d_{pillar}(μm)	$S_{pillar-pillar}$ (μm)	h_{pillar}(μm)
**Without pillars**	Control	-	-	-
**Square**	S120	128±12	122±5	132±13
**Hexagonal**	HE120	144±8	126±9	156±11
**Truncated cone**	TC120	128±6	129±8	138±9
**Cylindrical**	M120	149±10	125±1	127±11

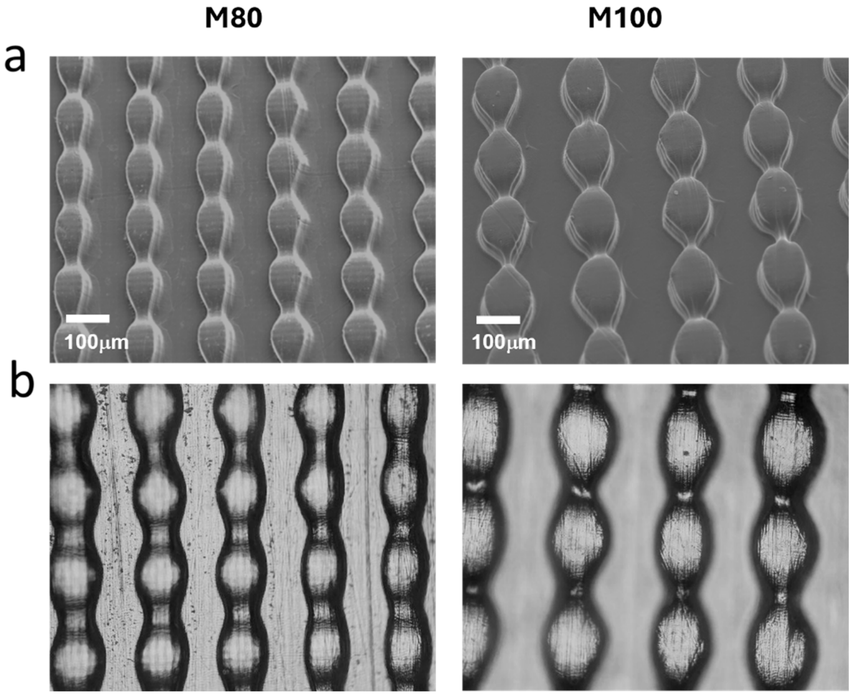

Figure S2. a) SEM images M80 and M100 taken at a tilting angle of 35° b) optical microscope images of M80 and M100 (100x).

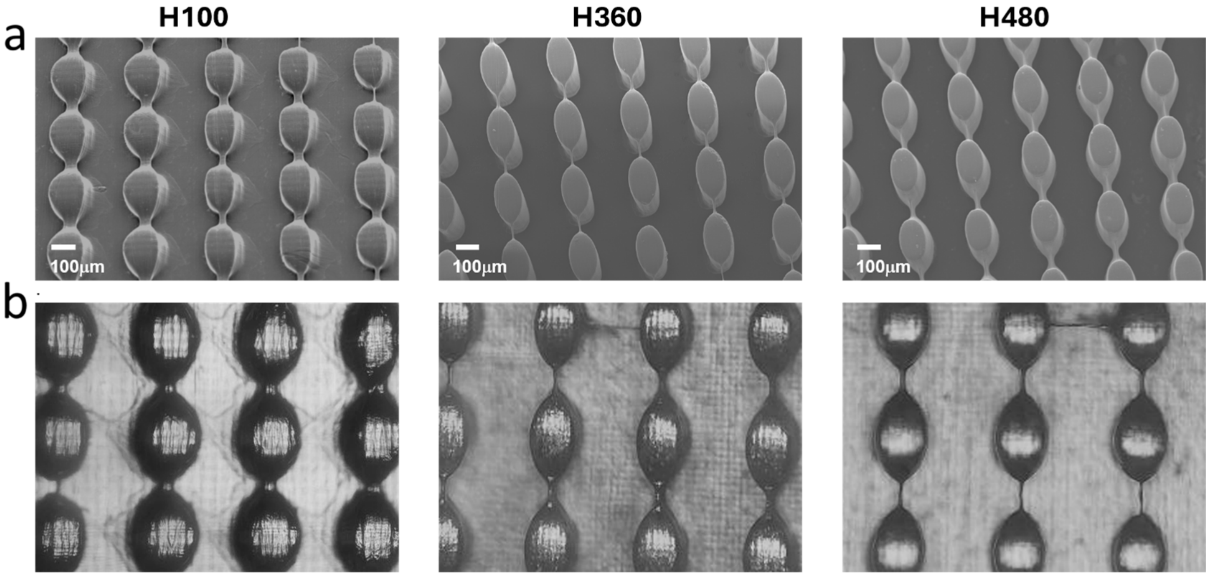

Figure S3. a) SEM images H100, H360 and H480 taken at a tilting angle of 35° b) optical microscope images of H100, H360 and H480 (100x).

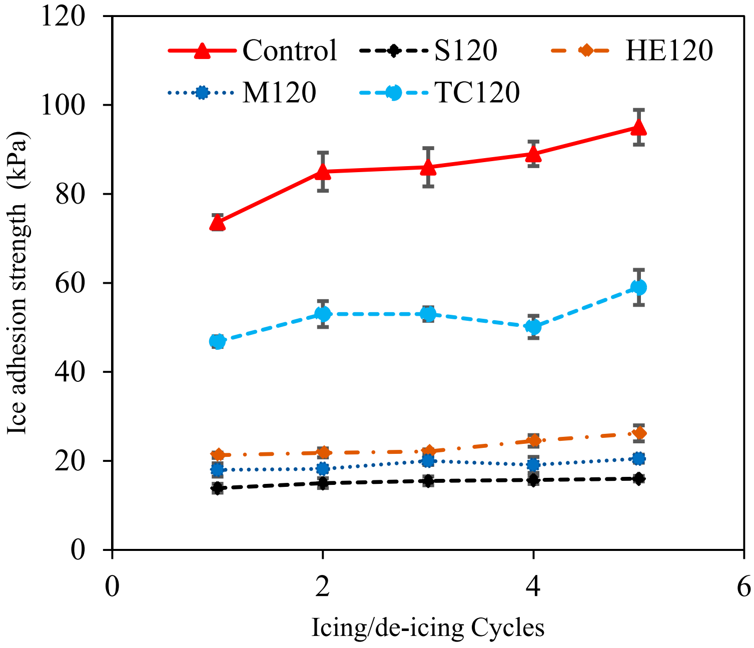

Figure S4. The ice adhesion strength of printed microstructures exposed to 5 icing/de-icing cycles obtained by push-off test.
